# First cycad seedling foliage from the fossil record and inferences for the Cenozoic evolution of cycads

**DOI:** 10.1098/rsbl.2019.0114

**Published:** 2019-07-10

**Authors:** Boglárka Erdei, Mario Coiro, Ian Miller, Kirk R. Johnson, M. Patrick Griffith, Vickie Murphy

**Affiliations:** 1 Botanical Department, Hungarian Natural History Museum, Könyves K. krt. 40, Budapest 1087, Hungary; 2 Department of Systematic and Evolutionary Botany, University of Zürich, Zollikerstrasse, 107 8008 Zürich, Switzerland; 3 Denver Museum of Nature and Science, 2001 Colorado Boulevard, Denver, CO 80205, USA; 4 Smithsonian National Museum of Natural History, 10th Street, Constitution Avenue North West, Washington, DC 20560, USA; 5 Montgomery Botanical Center, 11901 Old Cutler Road, Coral Gables, FL 33156, USA

**Keywords:** fossil seedling, cycad evolution, *Dioonopsis*, *Dioon*, Palaeocene

## Abstract

The morphology of the early ontogenetic stages of cycad foliage may help resolve the relationships between extinct to extant cycad lineages. However, prior to this study, fossil evidence of cycad seedlings was not known. We describe a compression fossil of cycad eophylls with co-occurring fully developed leaves of adult specimens from the early Palaeocene (*ca* 63.8 Ma) Castle Rock flora from the Denver Basin, CO, USA and assign it to the fossil genus *Dioonopsis* (Cycadales) based on leaf morphology and anatomy. The new fossil seedling foliage is particularly important because fully differentiated pinnate leaves of adult plants and the eophylls belong to the same species based on shared epidermal micromorphology, therefore, increasing the number of morphological characteristics that can be used to place *Dioonopsis* phylogenetically. Significantly, the seedling fossil has a basic foliage structure that is very similar to seedlings of extant cycads, which is consistent with a cycadalean affinity of *Dioonopsis*. Nevertheless, the set of morphological characters in the seedling and adult specimens of *Dioonopsis* suggests a distant relationship between *Dioonopsis* and extant *Dioon*. This indicates that extinct lineages of cycads were present and widespread during the early Cenozoic (Palaeogene) coupled with the subordinate role of extant genera in the Palaeogene fossil record of cycads.

## 1. Introduction

The evolution of cycads is a topic that fascinates an increasing number of researchers and avocational cycad enthusiasts. With 355 modern species in 10 genera [1], cycads have long been seen as relicts of a once flourishing and diverse group of plants [2]. Recent molecular phylogenetic studies, however, infer a late Palaeogene/Neogene origin and a late Miocene radiation for most extant genera [3,4] with only half of this diversity confirmed in the fossil record [5].

Fossils that show the early ontogenetic stages of plants may preserve important morphological characters to facilitate our understanding of the evolution of and relationships between extinct groups. These early stages, especially seedlings, are rarely fossilized. Among gymnosperms, seedlings have been reported only in few groups including scarce araucariaceous, taxodiaceous and pinaceous seedlings, and a few seedlings inferred as belonging to *Welwitschia*, ginkgophytes and glossopterids [6–14].

**Figure 1.**
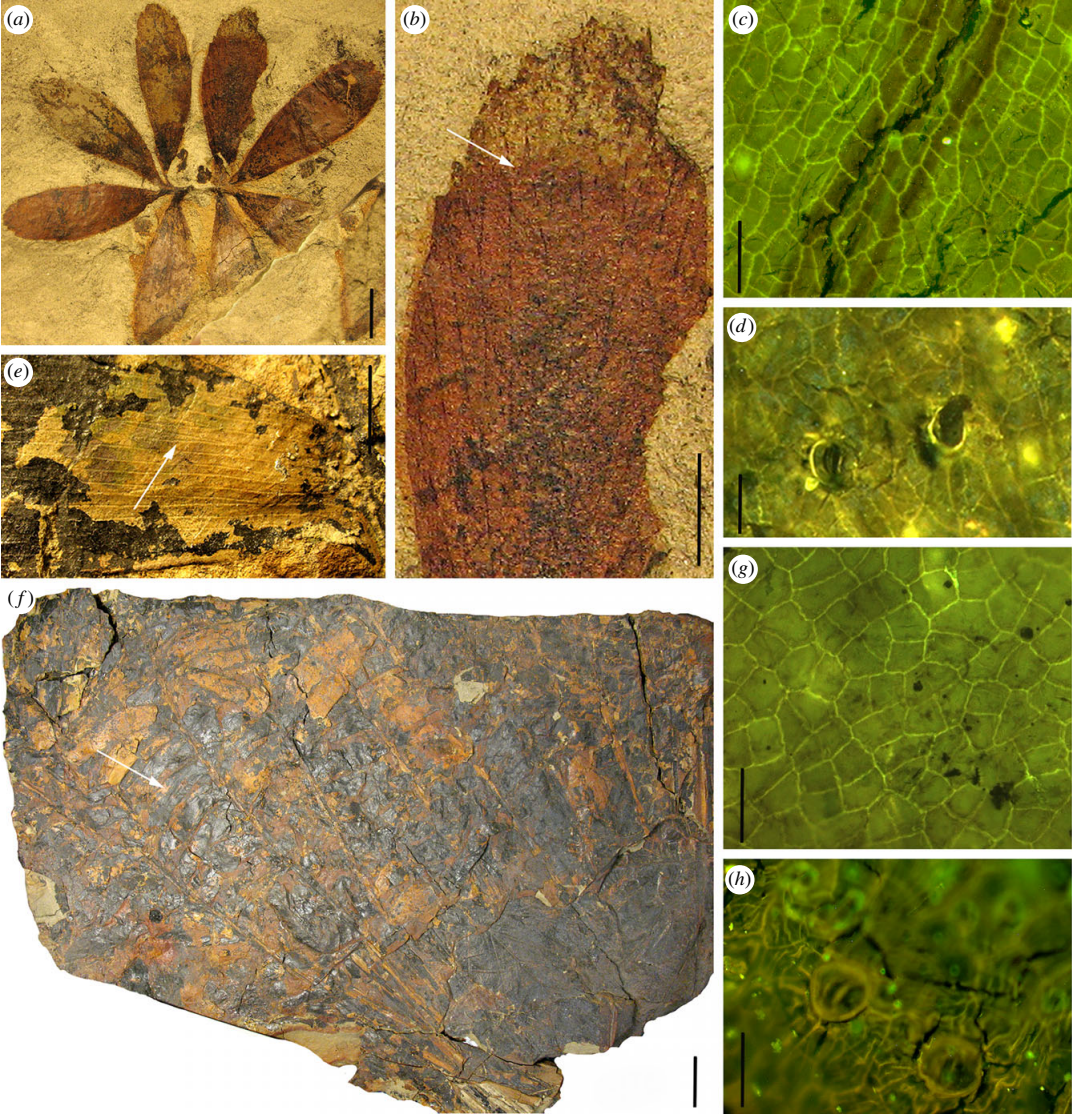
Eophylls and fully developed leaves of *Dioonopsis praespinulosa*. Eophylls (*a–d*). (*a*) Two eophylls (DMNH15662). (*b*) Enlargement of leaflet, arrow shows anastomosis of veins. (*c*) Epidermal cell pattern on the adaxial side of a leaf; note isodiametric anticlinal walls. (*d*) Cyclocytic stomata on the abaxial side of a leaf. Fully developed foliage of adult plants (*e–h*). (*e*) Leaflet showing a broad base where it attaches to the rachis; arrow shows N-shaped vein anastomoses (DMNH8993). (*f*) Fully developed leaves (leaf flush); arrow indicates the leaflet that provided the epidermal details shown in (*g,h*) (DMNH15683). (*g*) Epidermal cell pattern on the adaxial side of the leaf; note isodiametric anticlinal walls. (*h*) Cyclocytic stoma on the abaxial side of the leaf. Scale bars: (*a*) 1 cm, (*b*) 5 mm, (*c*) 50 µm, (*d*) 50 µm, (*e*) 1 cm, (*f*) 5 cm, (*g*) 50 µm, (*h*) 50 µm. (Online version in colour.)

Herein, we present the first fossil record of a cycad seedling found in close association with a leaf flush of an adult cycad plant of the same species (figure 1). The leaves of two ontogenetic stages, the eophylls (the first foliage) and the fully differentiated pinnate leaves of adult plants, are documented by macromorphological and epidermal features and assigned to the extinct genus, *Dioonopsis*. Based on the gross morphological similarity of the fully differentiated leaves, *Dioonopsis* has traditionally been assigned to the lineage leading to the extant cycad genus, *Dioon* [15,16], which lacks a fossil record. The new fossil seedling sheds light on the evolutionary relationship between *Dioonopsis* and extant cycads (*Dioon*).

## 2. Material and methods

Seedling foliage, a slab with numerous fully differentiated leaves of an adult plant, and many detached leaves assigned to cycads were reported among the fossils from the early Palaeocene (Danian; radiometrically dated at 63.84 ± 0.06 Ma) Castle Rock flora in the Denver Basin, CO, USA ([17–19]; electronic supplementary material, figures S1 and S2). The detailed investigation of these fossils is presented here for the first time. The specimens are housed in the palaeobotanical collection of the Denver Museum of Nature and Science, Denver, CO, USA (seedling fossil and its counterpart—DMNH15662, locality DMNH1200; fossil foliage of adult plants—DMNH15683 (leaf flush), DMNH15674, DMNH8993, locality DMNH1200).

Seedling macromorphology of extant cycad genera was examined for comparison (electronic supplementary material, table S1). Epidermal characters of the fossil foliage were studied using epifluorescence and transmitted light microscopy. The geology of the locality, the associated Castle Rock megaflora, detailed descriptions, inventories, storage and additional images of studied fossil cycad material and the methods applied for the investigation of epidermal characters on the fossils are discussed in the electronic supplementary material.

A matrix including 15 leaf morphological and cuticular characters, 10 characters modified from Martinez *et al*. [20] and five new characters (electronic supplementary material, note S1 and table S2), was scored for all extant genera of cycads and *Dioonopis* based on new character observations from the Castle Rock fossils and data from previous matrices and observations [21]. The most parsimonious placement of *Dioonopsis* on a phylogenetic tree based on [4] was tested by moving the taxon by hand in Mesquite [22] on a backbone phylogeny based on the results of the analyses of Salas-Leiva *et al*. [4], testing all possible placements of the fossil. Moreover, we conducted a maximum-parsimony analysis using PAUP v. 4.10 [23]. Most parsimonious trees were obtained using exhaustive enumeration of the trees compatible with the backbone constraint topology from Salas-Leiva *et al*. [4], and 1000 bootstrap replicates were run using the same constraint and a heuristic search strategy. A consensus network of the bootstrap trees was generated using SplitsTree [24] applying a 15% cut-off. We also conducted a Bayesian analysis using MrBayes v. 3.2.6 [25]. The Markov-k model with correction for variable characters and a gamma-distributed rate variation was applied. The topology from Salas-Leiva *et al*. [4] was used as a backbone constraint. Two independent runs with four chains (one cold, three heated) were run for 1 000 000 generations. After discarding 25% of each run as burn-in, the runs were combined and a consensus network was generated using SplitsTree applying a 15% cut-off.

## 3. Results and discussion

We assign the fossil foliage of adult specimens, including approximately 25 whole leaves in a single flush and many detached whole and partial leaves, to *Dioonopsis praespinulosa* described previously from the Palaeogene of Alaska [16] based on both macro- and micromorphology of the leaves (figure 1; electronic supplementary material, figures S3 and S4). The following characters support this taxonomic assignment: pinnate leaves; sub-opposite leaflets inserted with broad, decurrent bases laterally on rachis sides; a single order of parallel veins, which frequently dichotomize and locally form N-shaped anastomoses (i.e. neighbouring parallel veins traversed by a steep vein); isodiametric epidermal cells, cyclocytic stomata randomly arranged on the lower side of the leaves with four to seven radially arranged subsidiary cells. The 25 associated fully differentiated pinnate leaves (figure 1*f*), which presumably represents one leaf flush, indicate a plant with approximately 1-m-long leaves that grew in a bunch. This dimension is comparable to smaller or mid-sized members of extant cycads.

The fossil leaves are readily distinguished from other previously described Cenozoic cycads with pinnate leaves and parallel venation. The leaves of the extinct genus *Pseudodioon* from the Miocene of Turkey [26] differ by lacking vein anastomoses and having stomata in bands. Similarly, the extinct cycad *Pterostoma* from early Cenozoic floras in Australia [27] differs by having leaflets inserted on the rachis with slightly contracted bases and by an epidermal cell pattern with strongly undulate anticlinal walls, which contrasts the broad base of leaflets and basically straight anticlinal walls of *Dioonopsis*. Finally, the extinct foliage type, *Ctenis*, typically reported from Mesozoic floras, but also found in early Cenozoic floras of North America [28], is distinguished by having leaves with, in many cases, irregularly segmented leaflets with a high frequency of vein anastomoses. Compared to extant members of Cycadales, *Dioonopsis* is distinguished by its N-shaped vein anastomoses.

The eophylls are paripinnate having two pairs of leaflets, which bear teeth on the apical margin and frequently dichotomizing parallel veins (figure 1; electronic supplementary material, figure S3). Veins sporadically anastomose as well, forming N-shape cross-connections similar to those of fully differentiated pinnate leaves of adult plants. Based on shared venation characteristics (anastomoses) and epidermal features (figure 1; electronic supplementary material, figure S5), the eophylls represent the juvenile stage of *D. praespinulosa*. Detailed descriptions of the fossil specimens and additional photo documentation are given in the electronic supplementary material.

## 4. Nomenclature

*Dioonopsis praespinulosa* (Hollick) Erdei, Manchester et Kvaček, emend. nov. Erdei

**Figure 2.**
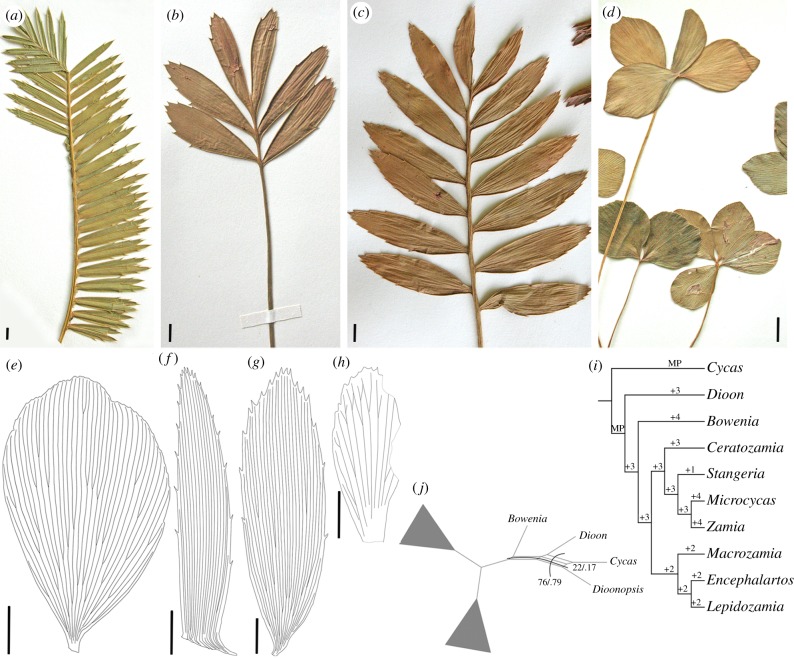
Comparison of eophylls of extant cycads and extinct *Dioonopsis* (*a–h*). (*a*) *Dioon merolae*. (*b*) *Encephalartos ferox*. (*c*) *Encephalartos transvenosus*. (*d*) *Zamia furfuracea*. Line drawings of cleared eophyll leaflets of extant cycads and extinct *Dioonopsis* showing venation details. (*e*) *Zamia furfuracea*. (*f*) *Dioon spinulosum*. (*g*) *Encephalartos hildebrandtii*. (*h*) *Dioonosis praespinulosa*. Scale bars: (*a–h*) 1 cm. (*i*) Number of steps needed for placing *Dioonopsis* on the molecular backbone topology. MP indicates the most parsimonious placements. (*j*) Consensus network showing support for the placement of *Dioonopsis* from the maximum-parsimony bootstrap and the Bayesian inference posterior probability. The two stronger splits are shown. (Online version in colour.)

Genus: *Dioonopsis* Horiuchi et Kimura, Review of Palaeobotany and Palynology, 51:217, 1987. Species: *Dioonopsis praespinulosa* (Hollick) Erdei, Manchester et Kvaček, *International Journal of Plant Sciences* **173**(1):83, figs 1–3*a*, 4 and 5, 2012, lectotype USNM38688b, figs 2*d–e*, 4*a,d*.

The emended diagnosis of *Dioonopsis praespinulosa*, given by Erdei, Manchester et Kvaček, *International Journal of Plant Sciences* **173**(1):83, is completed by adding the description of the eophylls (DMNH15662, Denver Museum of Nature and Science, Denver, CO, USA; figure 1*a–d*, electronic supplementary material, figures S3d,e and S5a–e).

*Emendation of the diagnosis*: Eophyll paripinnate, with few pairs of leaflets, leaflets obovate, apex slightly rounded, base decurrent, acute teeth on the apical one-third of lamina; 8–10 parallel veins entering leaflets, veins frequently dichotomize in the apical half of lamina, some veins sporadically anastomose forming N-shape; lamina hypostomatic, stomata cyclocytic, scattered, guard cells sunken, surrounded by four to seven subsidiaries, coronal rim formed around stomata, anticlinal walls isodiametric.

We surveyed the macromorphology of extant cycad seedlings and compared them to the seedling of *Dioonopsis praespinulosa* (figure 2; electronic supplementary material, table S1). The basic morphology of the *D. praespinulosa* eophylls closely resembles the eophylls of several extant cycads (e.g. *Encephalartos*), which is consistent with the cycadalean affinity of *Dioonopsis*. The eophylls of *D. praespinulosa* differ most strongly from those of *Cycas* and *Stangeria*, which have prominent midribs. In the majority of the extant members of Zamiaceae, which includes all extant cycad genera except *Cycas*, the eophylls and the fully differentiated leaves of adult plants of the same species have similar gross morphology. For instance, the eophyll and the fully differentiated leaves of adult specimens of most *Dioon* species are nearly indistinguishable. When differences do occur, they usually involve the shape of the leaflet (e.g. the eophylls of some *Zamia* and *Encephalartos* species, i.e. *E. ferox*, *Z. furfuracea*, are more ovate) or the frequency of teeth or spines on the leaflet margins as in some *Dioon* and *Encephalartos* species (e.g. *D. merolae*, *E. kisambo*). Finally, as seen in some *Ceratozamia*, *Encephalartos* and *Zamia* species, eophylls have considerably fewer leaflet pairs than the fully differentiated foliage of adult specimens has.

In contrast to many species of Zamiaceae, the seedling of *D. praespinulosa* has eophylls that differ markedly in leaflet shape, margin and number of leaflets from the fully differentiated leaves of adult plants. The morphological disparity of the seedling and the adult foliage of *Dioonopsis* may be compared to that of some *Encephalartos* species (figure 2).

The venation of both eophylls and the fully differentiated leaves of *Dioonopsis* differs significantly from all extant cycads. In particular, the veins anastomose to form an N-configuration—a consistent and distinctive character of both eophylls and fully differentiated foliage of adult plants of *Dioonopsis* [16,28]. Although this venation pattern does not appear in extant cycads, it is shared by fossil foliage assigned to Cycadales (e.g. *Ctenis* [28] and *Pterostoma* [27]) implying that an extinct lineage or lineages of cycads with similar venation patterns lived during the Mesozoic (*Ctenis*) and persisted into the Palaeogene (*Dioonopsis*, *Pterostoma*).

Among extant cycads, *Dioon* has been inferred to be related to the extinct *Dioonopsis* based on their shared gross morphology of leaves (parallel-veined leaflets with broad bases inserted laterally to the rachis) [15]. However, these genera are easily distinguished by leaf venation and epidermal characters (electronic supplementary material, table S2; [29]) and by the morphology of their seedlings. In fact, the contrasting morphological characters of the seedlings and adult specimens of *Dioonopsis* and *Dioon* suggest a distant relationship between these genera.

When considered in a phylogenetic context, our analysis indicates the placement of *Dioonopsis* outside Zamiaceae with both MP bootstrap and Bayesian posterior probability supporting this placement (figure 2). Similar results are obtained looking at the most parsimonious placement of the fossil, with only a placement close to *Stangeria*, being one step less parsimonious, which is due mostly to a reversal of typical zamiaceaous characters in the lineage leading to *Stangeria* (loss of elongate cells, irregular orientation of the stomata). A close relationship between *Dioonopsis* and *Dioon* retrieved in some previous phylogenetic analyses [20,21,30] is due mostly to the coding of *Dioonopsis* as having two layers of encircling cells (an autapomorphy of *Dioon* [29]), longitudinally oriented stomata (a potential synapomorphy of Zamiaceae [21]) and anticlinal plugs. These characters are clearly absent from all species of *Dioonopsis* and were probably originally miscoded based on some ambiguity in the original description [15].

The fossil genus and species, *Dioonopsis nipponica*, were established to accommodate cycad leaves with well-preserved epidermal details from the Palaeocene Noda Group of northeast Honshu, Japan [15]. Following the description of this material, Eocene cycad fossils from Alaska and California that were previously argued to be related to *Ceratozamia* and *Dioon* were found to belong to *Dioonopsis* [16]. The disjunct biogeographic distribution of *Dioonopsis* was interpreted as the consequence of spreading via the Beringia phytogeographic pathway [16]. The occurrence of *D. praespinulosa* in the Castle Rock flora (*ca* 63.8 Ma) further expands the North American distribution of *Dioonopsis* during the Palaeogene. Nevertheless, it does not offer additional clues about the place of origin of the genus because *Dioonopsis* appears roughly simultaneously, during the Palaeocene, in the fossil record of Japan and North America. However, the megafloras accompanying *Dioonopsis* do provide information on the ecological tolerance of the genus. In particular, the Castle Rock flora has been argued to be a high diversity, subtropical rainforest [31], whereas the Alaskan floras, although the Yakutat tectonic block was probably located further south [32], suggest more temperate conditions [16]. Assuming that, during the Palaeocene, *Dioonopsis* achieved higher palaeolatitudes (the Beringial passage) estimated at 75°–80° N, its adaptation to extreme light variations [16] was probably established, similarly to some extinct Southern Hemisphere cycads [33]. Considering these ecological factors, *Dioonopsis* had an ecological tolerance greater than any extant cycad genus.

Although there are rare examples of fossil cycads for which organs of the same genus or species were reported as associated (e.g. *Antarcticycas* [33]), the early and late ontogenetic stages of *Dioonopsis praespinulosa* that are preserved in the early Palaeocene Castle Rock flora are unprecedented. Even though there is an extensive record of extinct cycads [15,16,26–28,34–39], fossil data are commonly limited to adult leaves making a comparison to extant genera challenging. The co-occurring eophylls and fully developed adult foliage of *D. praespinulosa* allow for a much more complete analysis of *Dioonopsis* and its phylogenetic placement in Cycadales. Our work confirms that *Dioonopsis* belongs to an extinct lineage of cycads outside Zamiaceae. This pattern corroborates that today unknown cycad lineages flourished during the early Cenozoic (Palaeogene) [5]. The apparent scarcity of modern forms in both the Palaeogene and Mesozoic fossil record of cycads may be interpreted by a younger evolutionary radiation of modern cycads [3]. Nevertheless, the Cenozoic history of cycads is far from being resolved and requires additional fossil data.

## Supplementary Material

Material

## Supplementary Material

Methods

## Supplementary Material

Associated flora

## Supplementary Material

Descriptions

## Supplementary Material

Supplementary tables, Supplementary Note

## Data accessibility

Specimens studied are housed in the palaeobotanical collection of the Denver Museum of Nature and Science, Denver, CO. Full details of studied material, methodology and the associated Castle Rock flora are provided in the electronic supplementary material. The matrix used in this study is available on FigShare (https://figshare.com/s/f5a0068e7655e98b619e).

## References

[1] Calonje M, Stevenson DW, Stanberg L 2017 *The World List of Cycads* See http://www.cycadlist.org.

[2] Norstog KJ, Nichols TJ 1997 The biology of the cycads. Ithaca, NY: Cornell University Press.

[3] Nagalingum NS, Marshall CR, Quental TB, Rai HS, Little DP, Mathews S 2011 Recent synchronous radiation of a living fossil. Science 334, 796–799. (10.1126/science.1209926) 22021670

[4] Salas-Leiva DE, Meerow AW, Calonje M, Griffith MP, Francisco-Ortega J, Nakamura K, Stevenson DW, Lewis CE, Namoff S 2013 Phylogeny of the cycads based on multiple single-copy nuclear genes: congruence of concatenated parsimony, likelihood and species tree inference methods. Ann. Bot. 112, 1263–1278. (10.1093/aob/mct192) 23997230 PMC3806525

[5] Erdei B, Calonje M, Hendy A, Espinoza N 2018 A review of the Cenozoic fossil record of the genus *Zamia* L. (Zamiaceae, Cycadales) — evolution and biogeographic inferences. Bull. Geosci. 93, 185–204. (10.3140/bull.geosci.1671)

[6] Stockey RA 1975 Seeds and embryos of *Araucaria mirabilis*. Am. J. Bot. 62, 856–868. (10.1002/j.1537-2197.1975.tb14126.x)

[7] Stockey RA, Taylor TN 1978 On the structure and evolutionary relationships of the Cerro Cuadrado fossil conifer seedlings. Bot. J. Linn. Soc. 76, 161–176. (10.1111/j.1095-8339.1978.tb01504.x)

[8] Falder AB, Stockey RA, Rothwell GW 1999 *In situ* fossil seedlings of a *Metasequoia*-like taxodiaceous conifer from Paleocene river floodplain deposits of central Alberta, Canada. Am. J. Bot. 86, 900–902. (10.2307/2656710) 10371731

[9] Dörfelt H, Schmidt AR 2007 A conifer seedling with two herbicolous fungi from the Baltic amber forest. Bot. J. Linn. Soc. 155, 449–456 (10.1111/j.1095-8339.2007.00728.x)

[10] Rydin C, Mohr B, Friis EM 2003 *Cratonia cotyledon* gen. et. sp. nov.: a unique Cretaceous seedling related to *Welwitschia*. Biol. Lett. 270, 1–4. (10.1098/rsbl.2003.0044)) PMC1698015 12952628

[11] Dilcher DL, Bernardes-De-Oliveira ME, Pons D, Lott TA 2005 Welwitschiaceae from the lower Cretaceous of Northeastern Brazil. Am. J. Bot. 92, 1294–1310. (10.3732/ajb.92.8.1294) 21646150

[12] Bauer K, Grauvogel-Stamm L, Kustatscher E, Krings M 2013 Fossil ginkgophyte seedlings from the Triassic of France resemble modern *Ginkgo biloba*. BMC Evol. Biol. 13, 177 (10.1186/1471-2148-13-177) 23981276 PMC3765775

[13] Pant DD, Nautiyal DD 1987 *Diphyllopteris verticillata* Srivastava, the probable seedling of *Glossopteris* from the Palaeozoic of India. Rev. Palaeobot. Palynol. 51, 31–36. (10.1016/0034-6667(87)90017-0)

[14] Banerjee M 2000 *Deogharia nautiyalii* gen. et. sp. nov. *In situ* seedling of *Glossopteris* plant from Early Permian of Saharjuri Basin, Indian Lower Gondwana unlike *Diphyllopteris verticillata* Srivastava seedling. In Professor DD Nautiyal commemoration volume, *recent trends in botanical researches* (ed. Chauhan DK), pp. 157–164. Allahabad, India: Allahabad University.

[15] Horiuchi J, Kimura T 1987 *Dioonopsis* gen. et sp. nov., a new cycad from the Palaeogene of Japan. Rev. Palaeobot. Palynolol. 51, 213–225. (10.1016/0034-6667(87)90031-5)

[16] Erdei B, Manchester SR, Kvaček Z 2012 *Dioonopsis* Horiuchi et Kimura leaves from the Eocene of Western North America: a cycad shared with the Paleogene of Japan. Int. J. Plant Sci. 173, 81–95. (10.1086/662654)

[17] Ellis B, Johnson KR, Dunn RE 2003 Evidence for an *in situ* early Paleocene rainforest from Castle Rock, Colorado. Rocky Mount. Geol. 38, 73–100. (10.2113/gsrocky.38.1.173)

[18] Miller IM, Johnson KR, Ellis B 2007 A complete cycad plant from the Castle Rock rainforest: implications for the evolution and paleodistribution of American Zamiaceae. In Abstract 24th Annual Mid-Continent Paleobotanical Colloquium, Southern Methodist University, March 16–18.

[19] Kowalczyk JB et al. 2018 Multiple proxy estimates of atmospheric CO_2_ from an early Paleocene rainforest. Paleoceanogr. Paleoclim. 33, 1427–1438. (10.1029/2018PA003356)

[20] Martínez LCA, Artabe AEE, Bodnar J 2012 A new cycad stem from the Cretaceous in Argentina and its phylogenetic relationships with other Cycadales. Bot. J. Linn. Soc. 170, 436–458. (10.1111/j.1095-8339.2012.01300.x)

[21] Coiro M, Pott C 2017 *Eobowenia* gen. nov. from the Early Cretaceous of Patagonia: indication for an early divergence of *Bowenia*? BMC Evol. Biol. 17, 97 (10.1186/s12862-017-0943-x) 28388891 PMC5383990

[22] Maddison WP, Maddison DR 2015 Mesquite: a modular system for evolutionary analysis. Version 3.03 See http://mesquiteproject.org.

[23] Swofford DL 2003 PAUP*: phylogenetic analysis using parsimony (*and other methods). Version 4.0b10. (10.1111/j.0014-3820.2002.tb00191.x)

[24] Huson DH, Bryant D 2006 Application of phylogenetic networks in evolutionary studies. Mol. Biol. Evol. 23, 254–267. (10.1093/molbev/msj030) 16221896

[25] Ronquist F, Huelsenbeck JP 2003 MRBAYES 3: Bayesian phylogenetic inference under mixed models. Bioinformatics 19, 1572–1574. (10.1093/bioinformatics/btg180) 12912839

[26] Erdei B, Akgün F, Barone Lumaga MR 2010 *Pseudodioon akyoli* gen. et sp. nov., an extinct member of Cycadales from the Turkish Miocene. Plant System. Evol. 285, 33–49. (10.1007/s00606-009-0253-x)

[27] Hill RS 1980 Three new Eocene cycads from eastern Australia. Austr. J. Bot. 28, 105–122. (10.1071/BT9800105)

[28] Erdei B, Manchester SR 2015 *Ctenis clarnoensis* sp. n., an unusual cycadalean foliage from the Eocene Clarno Formation, Oregon. Int. J. Plant Sci. 176, 31–43. (10.1086/678467)

[29] Barone LMR, Coiro M, Truernit E, Erdei B, De Luca P. 2015 Epidermal micromorphology in *Dioon*: did volcanism constrain *Dioon* evolution? Bot. J. Linn. Soc. 179, 236–254. (10.1111/boj.12326)

[30] Hermsen EJ, Taylor TN, Taylor EL, Stevenson DW 2006 Cataphylls of the Middle Triassic cycad *Antarcticycas schopfii* and new insights into cycad evolution. Am. J. Bot. 93, 724–738. (10.3732/ajb.93.5.724) 21642136

[31] Johnson KR, Ellis B 2002 A tropical rainforest in Colorado 1.4 million years after the Cretaceous–Tertiary boundary. Science 296, 2379–2383. (10.1126/science.1072102) 12089439

[32] White T, Bradley D, Haeussler P, Rowley DB 2017 Late Paleocene–early Eocene paleosols and a new measure of the transport distance of Alaska's Yakutat Terrane. J. Geol. 125, 113–123. (10.1086/690198)

[33] Hermsen EJ, Taylor EL, Taylor TN 2009 Morphology and ecology of the *Antarcticycas* plant. Rev. Palaeobot. Palynol. 153, 108–123. (10.1016/j.revpalbo.2008.07.005)

[34] Hill RS, Pole MS 1994 Two new species of *Pterostoma* R.S. Hill from the Cenozoic sediments in Australasia. Rev. Palaeobot. Palynol. 80, 123–130. (10.1016/0034-6667(94)90097-3)

[35] Barthel M 1976 Farne und Cycadeen. Eozäne Floren des Geiseltales. Abhandlungen des Zentralen Geologischen Instituts 26, 439–490.

[36] Kvaček Z, Manchester SR 1999 *Eostangeria* Barthel (extinct Cycadales) from the Palaeogene of western North America and Europe. Int. J. Plant Sci. 160, 621–629. (10.1086/314152)

[37] Palamarev E, Uzunova K 1992 Beiträge zur Entwicklung der Cycadeen in der Tertiärflora Europas. Courier Forschungsinstitut Senckenberg 147, 287–293.

[38] Uzunova K, Palamarev E, Kvaček Z 2001 *Eostangeria ruzinciniana* (Zamiaceae) from the Middle Miocene of Bulgaria and its relationship to similar taxa of fossil *Eostangeria*, and extant *Chigua* and *Stangeria* (Cycadales). Acta Palaeobot. 41, 177–193.

[39] Wilf P, Stevenson DW, Cúneo NR 2016 The last Patagonian cycad, *Austrozamia stockeyi* gen. et sp. nov., early Eocene of Laguna del Hunco, Chubut, Argentina. Botany 94, 817–829. (10.1139/cjb-2016-0038)

