# Supplementary material for: First cycad seedling foliage from the fossil record and inferences for the Cenozoic evolution of cycads
Source: Biol Lett. 2019 Jul 10;15(7):20190114. doi: 10.1098/rsbl.2019.0114 (PMC6684986; doi:10.1098/rsbl.2019.0114)
Supplement: Material [file rsbl20190114supp1.docx]

**Electronic Supplementary Material**

**Collection material studied**

Fossil cycad specimens (seedling fossil and its counterpart, fossils of adult specimens) are housed in the palaeobotanical collection of the Denver Museum of Nature & Science, Denver, Colorado, USA.

Inventory numbers: seedling (Figure 1a–d, Figure S3d–e, Figure S5a–e) – DMNH15662, locality DMNH1200; adult leaf (Figure 1e, Figure S3b) – DMNH8993, locality DMNH1200; adult leaves (Figure 1f–h, Figure S3a, Figure S4) – DMNH15683, locality DMNH1200; adult leaf (Figure S3c) – DMNH15674, locality DMNH1200

The seedling fossil and its counterpart preserved both the abaxial and adaxial cuticles.

**Seedlings of modern cycads for comparison**

Modern cycads (seedlings) examined for comparisons were obtained mainly from living collections and herbaria of the Montgomery Botanical Center, Miami, Florida, USA and Fairchild Tropical Botanic Garden, Miami, Florida, USA.

*Bowenia spectabilis* Hook. ex Hook.f. – APII, Photo No.: rfk.9303, http://www.anbg.gov.au/cgi-bin/phtml?pc=rfk&pn=9303&size=3

*Ceratozamia* *kuesteriana* Regel – FTG61801;

*Ceratozamia* *matudae* Lundell – FTG62368;

*Ceratozamia* *mexicana* Brongn. – FTG67881;

*Ceratozamia* *miqueliana* H.Wendl. – FTG61800;

*Cycas* sp. – MBC486AAA (EX);

*Cycas* *clivicola* K.D.Hill – FTG99716;

*Cycas* *diannanensis* Z.T.Guan & G.D.Tao – MBC20130625 (EX);

*Cycas* *hoabinhensis* P.K.Lȏc & T.H.Nguyȩn – FTG101661;

*Cycas* *micholitzii* Dyer – MBC20130608 (EX);

*Cycas* *pectinata* Buch.-Ham. – FTG100424;

*Dioon* *merolae* De Luca, Sabato & Vázq.Torres – FTG67816;

*Dioon* *rzedowskii* De Luca, A.Moretti, Sabato & Vázq.Torres – MBC20140146 (EX);

*Dioon* *spinulosum* Dyer ex Eichler – MBC19830540AAA(parent) (EX);

*Encephalartos* *ferox* G.Bertol – FTG33273;

*Encephalartos* *gratus* Prain – MBC20140223 (EX);

*Encephalartos* *hildebrandtii* A.Braun & C.D.Bouché – MBC20140327 (EX);

*Encephalartos* kisambo Faden & Beentje – (KEW) K000986905 (http://specimens.kew.org/herbarium/K000986905)

*Encephalartos* *lanatus* Stapf & Burtt Davy – FTG9666;

*Encephalartos* *laurentianus* De Wild. – MBC20140010 (EX);

*Encephalartos* *transvenosus* Stapf & Burtt Davy – FTG16954;

*Encephalartos* *whitelockii* P.J.H.Hurter – FTBG198040 (EX);

*Lepidozamia* *hopei* Regel – APII, Photo No.: rfk.9570, http://www.anbg.gov.au/cgi-bin/phtml?pc=rfk&pn=9570&size=3

*Macrozamia* sp. – KEW, K001092661 (http://specimens.kew.org/herbarium/K001092661)

*Microcycas* *calocoma* (Miq.) A.D.C. – MBC20120373;

*Stangeria* *eriopus* (Kunze) Baill. – (KEW) K001107022 (http://specimens.kew.org/herbarium/K001107022)

*Zamia* sp. – MBC20020542AAA(parent) (EX); MBC20050312AAA(parent) (EX); MBC20030212AAA(parent) (EX);

*Zamia* *erosa* O.F.Cook & G.N.Collins – MBC20130747 (EX);

*Zamia* *furfuracea* L.f. – MBC2004068AAA(parent) (EX); FTG103312; FTG140759; FTG83199; FTG120409;

*Zamia* *herrerae* S.Calderón & Standl. – MBC20120219;

*Zamia* *inermis* Vovides, J.D.Rees & Vázq. Torres – MBC92143D(parent) (EX);

*Zamia* *integrifolia* L.f. – MBC20130745 (EX);

*Zamia* *prasina* W.Bull – MBC20140328 (EX);

*Zamia* *skinneri* Warsz. ex A.Dietr. – MBC20110525;

*Zamia* *vazquezii* D.W.Stev., Sabato & De Luca – MBC2000137A (EX);

**Abbreviations of living collections and herbaria**:

APII – Australian Plant Image Index, Australian National Botanic Gardens, Australian National Herbarium

EX – Exsiccata specimens of modern seedlings are stored in the herbarium of the Hungarian Natural History Museum (Budapest), Hungary.

FTBG – Fairchild Tropical Botanic Garden, Florida, USA

FTG – Herbarium of the Fairchild Tropical Botanical Garden, Florida, USA

KEW – Royal Botanic Gardens KEW, KEW Databases

MBC – Montgomery Botanical Center, Florida, USA
