# Supplementary material for: First cycad seedling foliage from the fossil record and inferences for the Cenozoic evolution of cycads
Source: Biol Lett. 2019 Jul 10;15(7):20190114. doi: 10.1098/rsbl.2019.0114 (PMC6684986; doi:10.1098/rsbl.2019.0114)
Supplement: Methods [file rsbl20190114supp2.docx]

**Electronic Supplementary Material**

**Methods**

**Sampling for light and fluorescence microscopy**

Epidermal characters were studied using epifluorescence and transmitted light microscopy on flakes of cuticle in the case of the fossil adult foliage and on in-situ cuticle from the fossil seedling.

Cuticle fragments were removed from the fossil adult foliage and cleaned in HF, washed, and examined dry or in water on a glass slide. Epidermal characters of the seedling were studied intact on the specimen.

Leaf material of extant seedlings for comparison (cleared leaflets) was treated with an aquaeous solution of KOH (5%) for several hours, as required, at room temperature, then washed in water.

Epidermal characters were studied using a Zeiss Axiophot microscope (Florida Museum of Natural History, Gainesville) and a Nikon Eclipse E600 microscope (Hungarian Natural History Museum, Budapest).

**Photography**

Photos of epidermal characters were taken with an Axiocam digital camera attached to Zeiss Axiophot microscope (Florida Museum of Natural History, Gainesville) and a QImaging Micropublisher 3.3 RTV camera attached to Nikon Eclipse E600 microscope (Hungarian Natural History Museum, Budapest).
