# Supplementary material for: First cycad seedling foliage from the fossil record and inferences for the Cenozoic evolution of cycads
Source: Biol Lett. 2019 Jul 10;15(7):20190114. doi: 10.1098/rsbl.2019.0114 (PMC6684986; doi:10.1098/rsbl.2019.0114)
Supplement: Associated flora [file rsbl20190114supp3.docx]

**Electronic Supplementary Material**

**The associated Castle Rock flora**

The fossil cycad seedling (eophylls) and fully developed (adult) foliage was collected near Castle Rock, in the Denver Basin, Colorado, USA. The Castle Rock flora (DMNH loc. 1200 and associated later localities) was discovered in the late 1990’s during the realignment of a railroad overpass on Interstate 25. The locality occurs within the city limits of Castle Rock at the western base of the butte in the middle of the town. The Denver Basin (Figure S1) is a synorogenic basin formed during the Laramide Orogeny beginning at approximately 70 Ma in the Southern Rocky Mountains (Raynolds and Johnson, 2002). The terrestrial Laramide-age (Maastrichtian through Early Eocene) strata in the basin host abundant fossil plants.

.

The Castle Rock flora (63.84 ± 0.06 Ma as dated using U-Pb geochronology [Kowalczyk et al., 2018]) occurs in the Denver Formation and is composed of leaves from a forest that grew approximately 2.2 Ma after the Cretaceous-Paleogene (K-Pg) boundary (see Clyde et al., 2016 for an estimated K-Pg boundary date Denver Basin). The flora is unusual in that it shows very high plant species richness as recorded by leaf morphotypes (analogous to species) immediately following the K-Pg boundary (Johnson and Ellis, 2002) with 93 taxa documented from a collection of 1,030 fossil leaf specimens (Ellis et al., 2003). Moreover, the leaf physiognomy of the flora exhibits aspects of rainforests (examples include drip tips, large leaves, high diversity, and an estimated mean annual temperature in excess of 20º C [Johnson and Ellis, 2002; Ellis et al., 2003]). Aspects of the flora also indicate decoupled foodweb recovery following the extinction. In particular, insect damage types on leaves is low when compared to floral diversity at Castle Rock or insect damage types on leaves in other similarly aged floras (Wilf et al., 2006).

The high plant diversity at Castle Rock is interpreted as being the result of 1) intensified orographic precipitation; 2) proximity to the Paleocene mountain front in the basin leading to increased niche space; and 3) taphonomic conditions wherein successive overbank deposits buried a series of mature forest floors (Ellis et al., 2003; see also Ellis and Johnson, 2013). The cycad seedling and adult plant, which is represented by a leaf flush with approximately 25 complete leaves near a possible *in-situ* stem, are indicative of rapid burial of an established forest floor (Figure S2; see Ellis et al., 2003).

**References**

Clyde WC, Ramezani J, Johnson KR, Bowring SA, Jones MM. 2016 Direct high-precision U-Pb geochronology of the end-Cretaceous extinction and calibration of Paleocene astronomical timescales. *Earth and Planetary Science Letters* **452**, 272–280.

Ellis B, Johnson KR, Dunn RE. 2003 Evidence for an in situ early Paleocene rainforest from Castle Rock, Colorado. *Rocky Mountain Geology* **38**, 73–100.

Ellis B, Johnson KR. 2013. Comparison of leaf samples from mapped tropical and temperate forests: Implications for interpretations of the diversity of fossil assemblages. *Palaios* **28(3)**, 163–177.

Johnson KR, Ellis B. 2002 A tropical rainforest in Colorado 1.4 million years after the Cretaceous-Tertiary boundary. *Science* **296**, 2379–2383.

Kowalczyk JB, Royer DL, Miller IM, Anderson CW, Beerling DJ, Franks PJ, Grein M, Konrad W, Roth-Nebelsick A, Bowring SA, Johnson KR, Ramezani J. 2018 Multiple proxy estimates of atmospheric CO2 from an early Paleocene rainforest: *Paleoceanography and Paleoclimatology* **33**(12), doi: 10.1029/2018PA003356.

Raynolds RG, Johnson KR. 2002 Upper Cretaceous and Tertiary stratigraphy of the Denver Basin, Colorado. *Rocky Mountain Geology* **37**, 111–134.

Wilf P, Labandeira CC, Johnson KR, Ellis B. 2006 Decoupled plant and insect diversity after the End-Cretaceous extinction. *Science* **313**, 1112–1115.

**Figure S1**

Geologic map of the Denver Basin showing the location of the Castle Rock flora and synorogenic Laramide formations in the basin. The Castle Rock flora occurs in the Paleocene strata of the Denver Formation. The D1 and D2 sequences refer to the time-stratigraphic synorogenic sequences of sediment that are nearly analogous to the Denver and Dawson Formations, respectively (figure modified from Raynolds and Johnson, 2002).


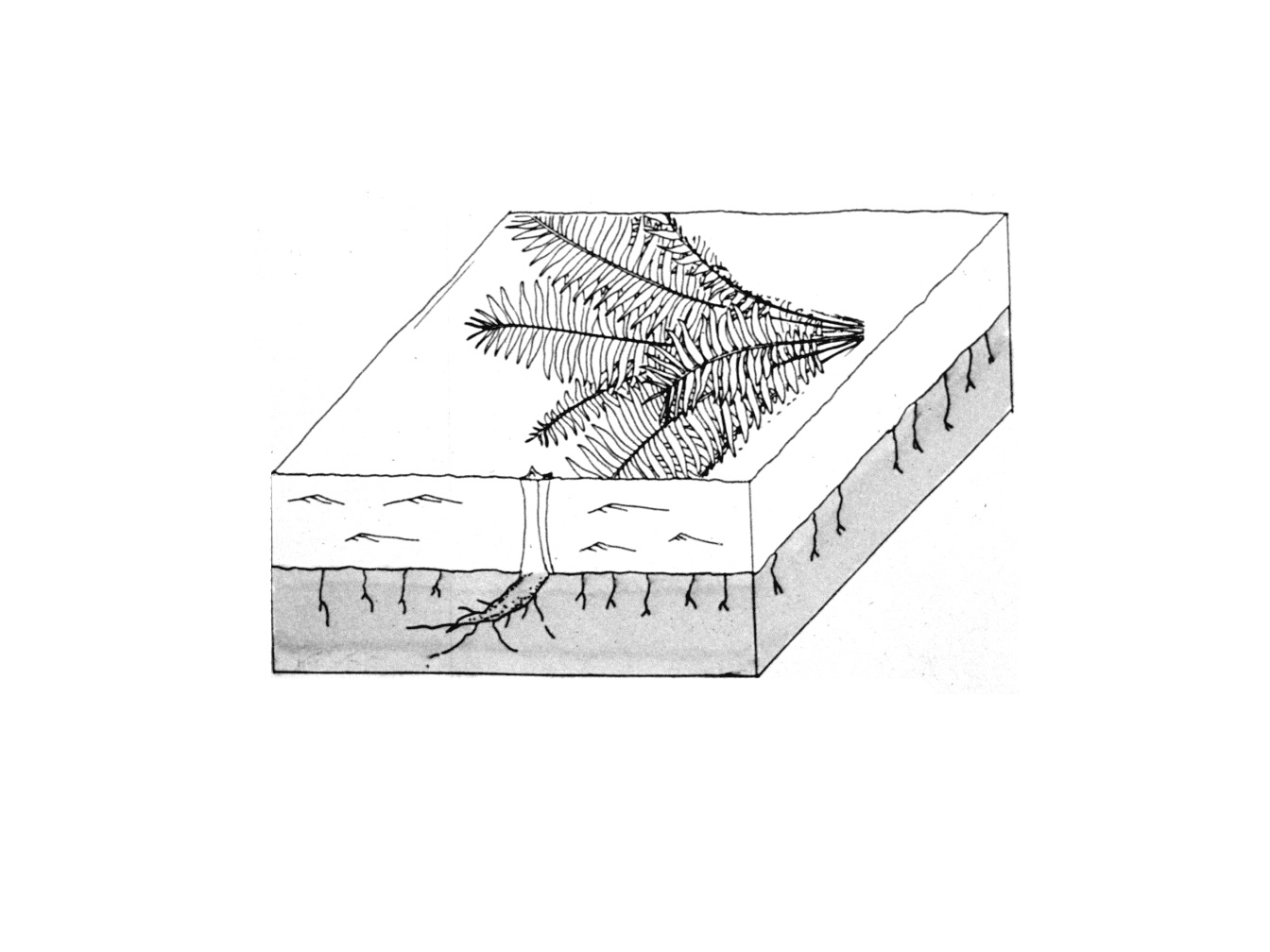


**Figure S2**

Line drawing showing the position of the “leaf flush” of the *Dioonopsis* adult plant and a possible *in-situ* corm as found in quarry (drawn by Kirk Johnson).
