# Supplementary material for: First cycad seedling foliage from the fossil record and inferences for the Cenozoic evolution of cycads
Source: Biol Lett. 2019 Jul 10;15(7):20190114. doi: 10.1098/rsbl.2019.0114 (PMC6684986; doi:10.1098/rsbl.2019.0114)
Supplement: Descriptions [file rsbl20190114supp4.docx]

Spermatophyta.

Order—Cycadales Dumort.

Family—?

Genus—*Dioonopsis* Horiuchi et Kimura

Species— *Dioonopsis* *praespinulosa* (Hollick) Erdei, Manchester et Kvaček emend. Erdei

Description. Fully differentiated leaves are pinnate and may reach 1 metre in length (Figure S3a–c). Leaflets show variable size (length and width ranging up to 15 cm and 2.5 cm respectively) depending on their point of origin on the leaf. Leaflets are sub-opposite, broadly and laterally attached to the rachis in one plane. Leaflet bases are decurrent and apices are acute. Shape of leaflets is narrow lanceolate to linear. Leaflets are mostly entire margined, few teeth occur. There are 15–20 parallel veins, which commonly dichotomize and anastomose in some cases forming a characteristic N-shape. Additional foliar characters of the species are given in Erdei et al. (2012).

The seedling eophylls are oriented in a way suggesting that these belong to one individual. However, they might represent separate individuals since their petioles and underground parts were not observed. Eophylls are paripinnate, with four leaflets on each eophyll. Leaflets are 3.5–4 cm long, 1.1–1.3 cm wide, slightly obovate with rounded apex and decurrent base (Figure S3c–d). The leaflet margins are entire on the lower two-thirds of the lamina and toothed on the apical one-third of the lamina. Teeth are acute. Venation is open, parallel, with 8-10 veins entering the leaflets. Veins commonly dichotomize in the apical half of the lamina. Few anastomoses of veins form an N-shape. Termination of veins is not clearly observable. Some veins seem to end in apical teeth but the very end of veins is not preserved.


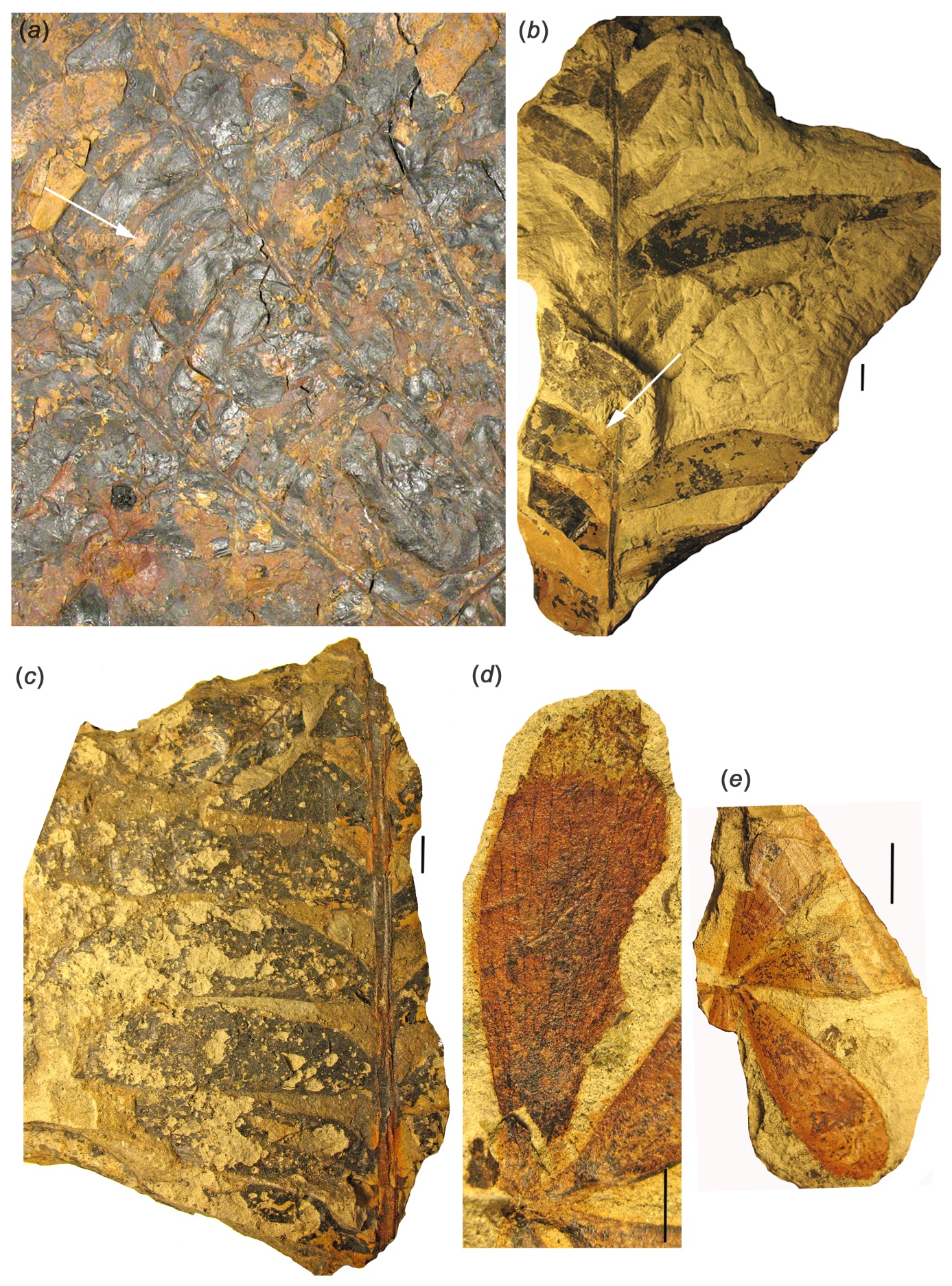


**Figure S3**

Fully differentiated leaves of adult specimens (a–c) and eophyll (d–e) of *Dioonopsis* *praespinulosa* from the Castle Rock flora, Denver Basin, Colorado.

(a) Detail of fully differentiated foliage shown on Figure 1(f), Nearly whole leaves probably representing one flush. The medial and ?basal sections are preserved, arrow indicates part of a leaflet providing epidermal details (DMNH 15683; scale bar 5 cm). (b) Fully differentiated leaf, detached specimen, arrow indicates leaflet shown on Figure 1(e) (DMNH 8993; scale bar 1 cm). (c) Medial section of a fully differentiated leaf (DMNH 15674; scale bar 1 cm); (d) Enlarged leaflet of eophyll shown on Figure 1(a) (DMNH 15662; scale bar 0.5 cm); (e) Eophyll, counterpart of fossil shown on Figure 1(a) (DMNH 15662; scale bar 1 cm);

The eophylls of the seedling and the leaves of the adult plant display similar epidermal features (see also [14]) with minor differences (Figure S4, S5). Lamina is hypostomatic. Stomata are cyclocytic (amphicyclic), scattered, and randomly arranged. Guard cells are sunken and surrounded by radially arranged subsidiaries (four to seven). A cutinized coronal rim (diameter 30–50 µm) is formed around stomata. Ordinary epidermal cells are isodiametric, measuring 20–55 µm. Angular trichome bases are frequently present on abaxial side of adult foliage but were not observed in the case of the eophylls. Abaxial cuticle of the adult foliage shows striations not observed in the case of the eophylls.

Minor differences in epidermal details may be attributable to the different ontogenetic stages. A different display of epidermal characteristics in adult and juvenile leaves (e.g. stomatal distribution amphistomatic/hypostomatic, occurrence or absence of papillae and crystalliferous cells, etc.) were recognized in extant *Macrozamia* species (Carpenter et al., 1991).

Nomenclature. The seedling and the associated fully developed leaves are both related to the same species based on shared epidermal anatomy. Therefore we retain a single fossil-taxon for the two life history stages (complying with Shenzhen Code, Art.1.2.).


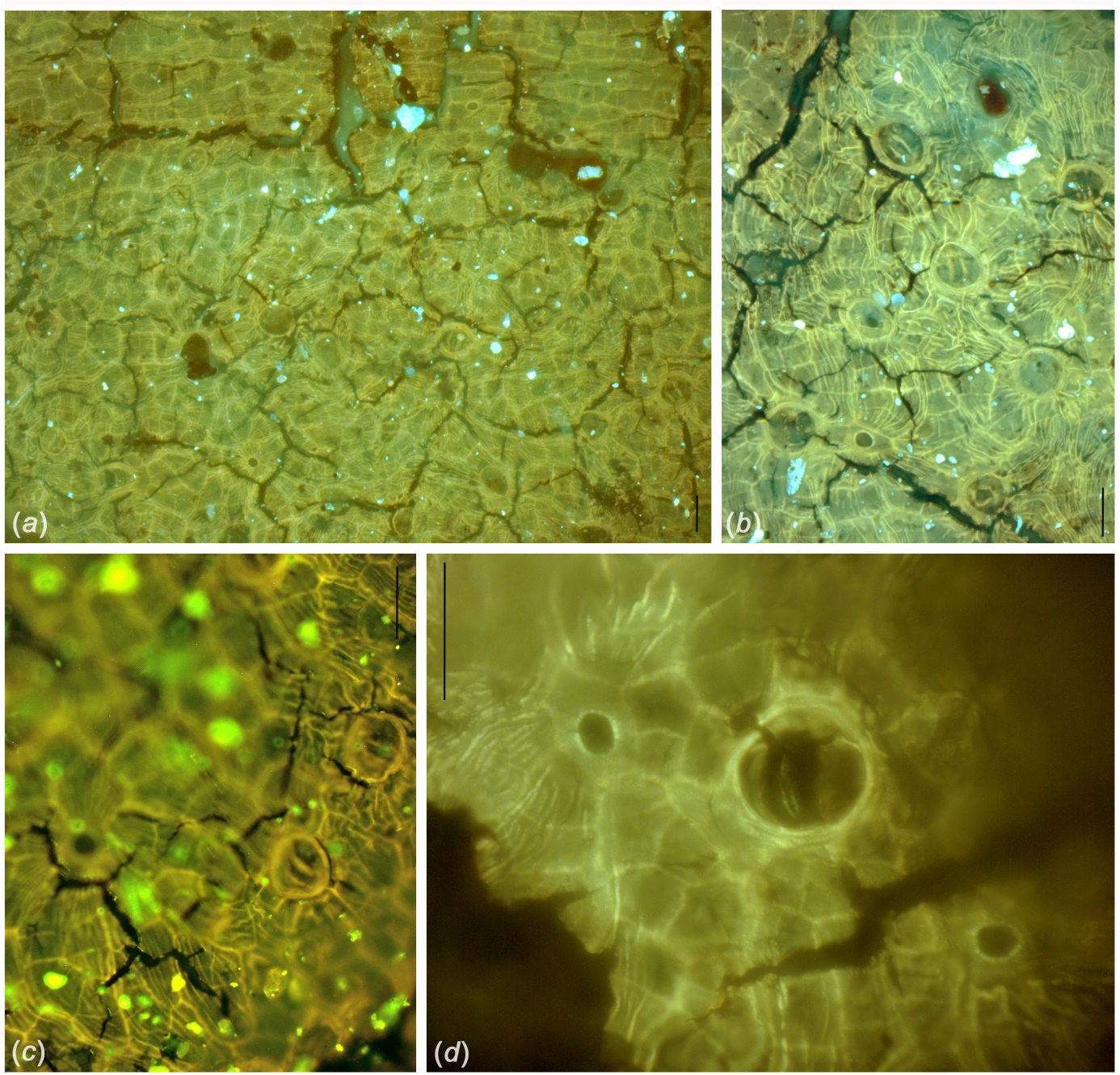


**Figure S4**

Epidermal details of *Dioonopsis* *praespinulosa* adult foliage (DMNH 15683) from the Castle Rock flora, Denver Basin, Colorado; scale bars 50 µm.

(a) Isodiametric cells and elongated cells along vein on adaxial side of leaflet. (b) Abaxial side of leaflet showing scattered cyclocytic stomata and trichome bases, cuticle is striated. (c) Abaxial side of leaflet showing cyclocytic stomata, cuticle is striated. (d) A stomatal apparatus and two trichome bases in higher magnification, striations are observable.


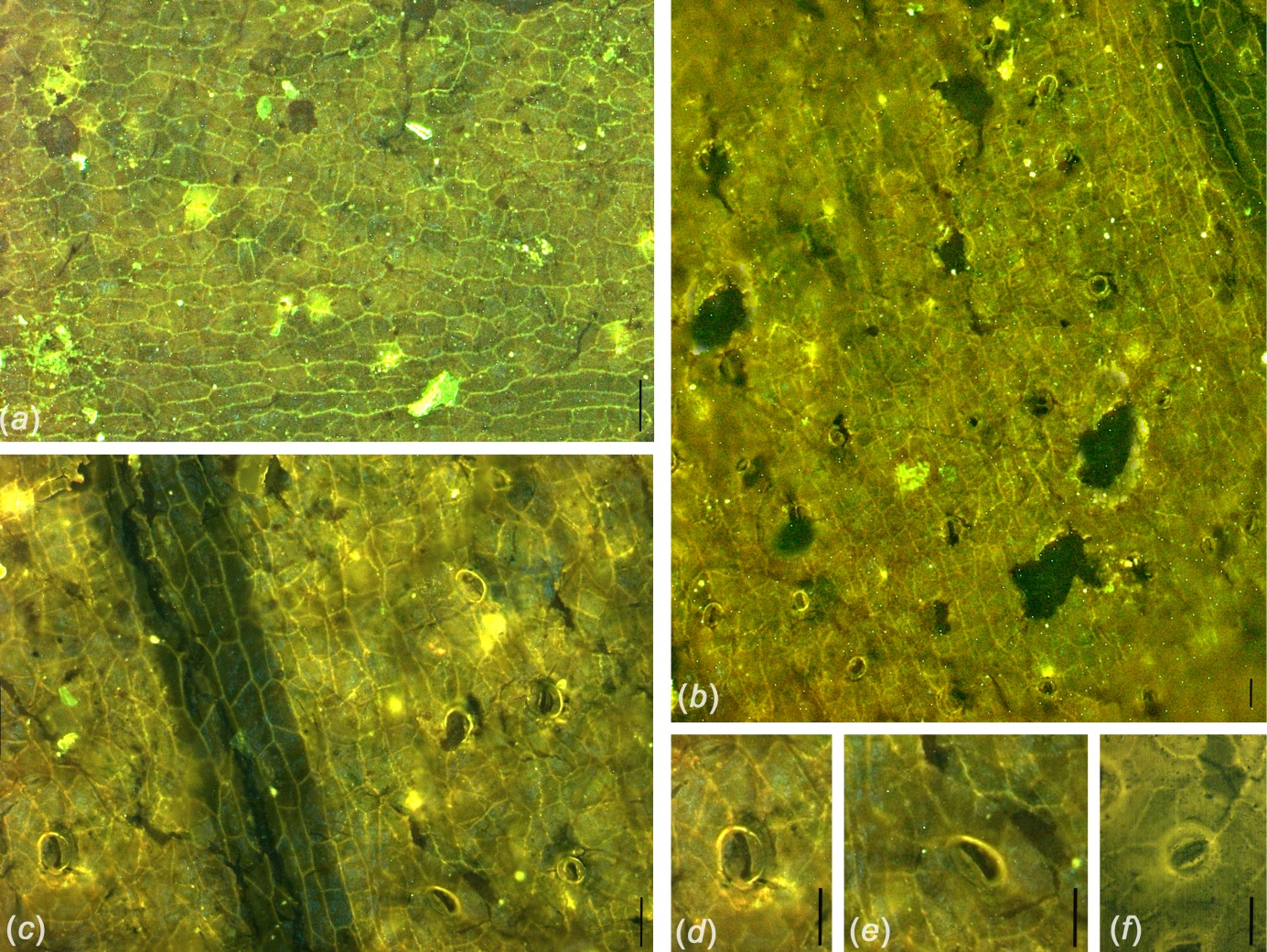


**Figure S5**

Epidermal details of eophylls of *Dioonopsis* *praespinulosa* from the Castle Rock flora, Denver Basin, Colorado (a–e) and adult foliage of *Dioonopsis* *praespinulosa* from Hamilton Bay, Alaska (f); scale bars 50 µm.

(a) Adaxial side of eophyll leaflet showing isodiametric cell pattern (DMNH 15662). (b) Abaxial side of eophyll leaflet showing scattered, cyclocytic stomata (DMNH 15662). (c) Detail of (b) in higher magnification, cells along vein are slightly elongated. (d) – (e) Details of (b) showing stomata in higher magnification. (f) Abaxial cuticle of the lectotype of *Dioonopsis* *praespinulosa* showing a stoma in higher magnification (lectotype, USNM 38688b).

**References**

Carpenter RJ. 1991 *Macrozamia* from the early Tertiary of Tasmania and a study of the cuticles of extant species. Australian Systematic Botany 4, 433–444.

Erdei B, Manchester SR, Kvaček Z. 2012 *Dioonopsis* Horiuchi et Kimura leaves from the Eocene of Western North America: a cycad shared with the Paleogene of Japan. *International Journal of Plant Sciences* **173**, 81–95.
