# Supplementary material for: First cycad seedling foliage from the fossil record and inferences for the Cenozoic evolution of cycads
Source: Biol Lett. 2019 Jul 10;15(7):20190114. doi: 10.1098/rsbl.2019.0114 (PMC6684986; doi:10.1098/rsbl.2019.0114)
Supplement: Supplementary tables, Supplementary Note [file rsbl20190114supp5.docx]

| Genus | leaf shape | leaflet number | leaflet shape | venation | anastomoses  of veins |
| --- | --- | --- | --- | --- | --- |
| *Cycas* | pinnate | comparable to adult foliage | similar to adult foliage | midrib | --- |
| *Dioon* | pinnate | comparable to adult foliage | similar to adult foliage | parallel | marginal (terminal) anastomoses |
| *Macrozamia* | pinnate | comparable to adult foliage | similar to adult foliage | parallel | marginal (terminal) anastomoses |
| *Lepidozamia* | pinnate | comparable to adult foliage | similar to adult foliage | parallel | marginal (terminal) anastomoses |
| *Encephalartos* | pinnate | few pairs of leaflets | obovate/ovate, different from adult foliage | parallel | marginal (terminal) anastomoses |
| *Ceratozamia* | pinnate | few pairs of leaflets | similar to adult foliage | parallel | no anastomoses |
| *Stangeria* | pinnate | comparable to adult foliage | similar to adult foliage | midrib | occasional marginal anastomoses |
| *Zamia* | pinnate | few pairs of leaflets | different from adult foliage in some species | parallel | no anastomoses |
| *Microcycas* | pinnate | comparable to adult foliage | similar to adult foliage | parallel | no anastomoses |
| *Bowenia* | pinnate | comparable to adult foliage | similar to adult foliage | parallel | no anastomoses |
| *Dioonopsis* | pinnate | few pairs of leaflets | obovate, different from adult foliage | parallel | N-shape anastomoses over lamina surface |

Table S1. Summary of eophyll macromorphology of extant cycads and extinct *Dioonopsis*.

| Genus | Ch.1. | Ch.2. | Ch.3. | Ch.4. | Ch.5. | Ch.6. | Ch.7. | Ch.8. |
| --- | --- | --- | --- | --- | --- | --- | --- | --- |
| *Cycas* | 0 | 0 | 0 | 0 | 1 | 0 | 0 | 0 |
| *Dioon* | 0 | 0 | 0 | 0 | 0 | 1 | 0 | 1 |
| *Macrozamia* | 0 | 0 | 0 | 1 | 0 | 1 | 0 | 1 |
| *Lepidozamia* | 0 | 1 | 0 | 1 | 0 | 1 | 0 | 1 |
| *Encephalartos* | 0 | 0 | 0 | 0 | 0 | 1 | 0 | 1 |
| *Ceratozamia* | 0 | 0 | 1 | 0 | 0 | 1 | 0 | 0 |
| *Stangeria* | 0 | 0 | 0 | 0 | 1 | 1 | 0 | 0 |
| *Zamia* | 0 | 0 | 1 | 0 | 0 | 1 | 0 | 0 |
| *Microcycas* | 0 | 0 | 1 | 0 | 0 | 1 | 0 | 0 |
| *Bowenia* | 1 | 0 | 1 | 0 | 0 | 1 | 0 | 0 |
| *Dioonopsis* | 0 | 0 | 0 | 0 | 0 | 1 | 1 | 1 |

| Taxon | Ch.9. | Ch.10. | Ch.11. | Ch.12. | Ch.13. | Ch.14. | Ch.15. |
| --- | --- | --- | --- | --- | --- | --- | --- |
| *Cycas* | 0 | 0 | 0 | 1 | 0 | 0 | 0 |
| *Dioon* | 1 | 1 | 1 | 2 | 1 | 0 | 0 |
| *Macrozamia* | 1 | 1 | 1 | 1 | 1 | 1 | 0 |
| *Lepidozamia* | 1 | 1 | 1 | 1 | 1 | 1 | 0 |
| *Encephalartos* | 1 | 1 | 1 | 1 | 1 | 1 | 0 |
| *Ceratozamia* | 1 | 1 | 1 | 1 | 1 | 1 | 0 |
| *Stangeria* | 0 | 1 | 0 | 1 | 1 | 0 | 1 |
| *Zamia* | 1 | 1 | 1 | 1 | 1 | 1 | 0 |
| *Microcycas* | 1 | 1 | 1 | 1 | 1 | 0 | 0 |
| *Bowenia* | 1 | 1 | 1 | 0 | 1 | 0 | 1 |
| *Dioonopsis* | 0 | 0 | 0 | 1 | 0 | 1 | 0 |

Table S2. Character matrix including 15 leaf and cuticular characters for all extant genera of cycads and *Dioonopis* based on new observations as well as previous matrices and observations. Explanation of Characters see in Note S1.

|  | **Extant only** | | **With *Dioonopsis*** | |
| --- | --- | --- | --- | --- |
| **Char** | **CI** | **RI** | **CI** | **RI** |
| 1 | uninformative | uninformative | uninformative | uninformative |
| 2 | uninformative | uninformative | uninformative | uninformative |
| 3 | 0.5 | 0.5 | 0.5 | 0.5 |
| 4 | 0.5 | 0 | 0.5 | 0 |
| 5 | 0.5 | 0 | 0.5 | 0 |
| 6 | uninformative | uninformative | uninformative | uninformative |
| 7 | uninformative | uninformative | uninformative | uninformative |
| 8 | 0.5 | 0.75 | 0.33 | 0.5 |
| 9 | 0.5 | 0.5 | 0.5 | 0.5 |
| 10 | 1 | 1 | 1 | 1 |
| 11 | 0.5 | 0.5 | 0.5 | 0.5 |
| 12 | uninformative | uninformative | uninformative | uninformative |
| 13 | 1 | 1 | 1 | 1 |
| 14 | 0.33 | 0.5 | 0.25 | 0.25 |
| 15 | 0.5 | 0 | 0.5 | 0 |

**Table S3.** Consistency index (CI) and retention index (RI) for the 15 characters optimized on the Salas-leiva et al. (2013: [4]) tree (Left) and the tree with *Dioonopsis* in its most parsimonious placement (Right).

**Note S1. Description of the characters employed in phylogenetic analyses.**

Character 1: Leaf shape (0) pinnate (1) bipinnate. Modified from character 24 of Martinez et al. 2012.

Character 2: Leaflet attachment (0) medial (1) adaxial. Modified from character 26 of Martinez et al. 2012.

Character 3: Leaflet articulation (0) decurrent (1) articulate. Modified from character 27 of Martinez et al. 2012.

Character 4: Acroscopic callus (0) absent (1) present. Character 29 of Martinez et al. 2012.

Character 5: Midrib (0) absent (1) present. New character to separate *Cycas* and *Stangeria* from all other genera without a midrib. Zamia is coded with midrib absent, given the nested position of *Zamia restrepoi* within the genus (Calonje et al. 2019).

Character 6: Venation (0) single veins (1) parallel veins. Modified from character 30 of Martinez et al. 2012.

Character 7: N-anastomoses (0) absent (1) present. New character that codes for the presence of N-anastomoses in *Dioonopsis.*

Character 8: Terminal vein fusion (0) absent (1) present. This character substitutes character 33 of Martinez et al. 2012, that does not distinguish between terminal vein fusion and N-anastomoses.

Character 9: Stomatal orientation (0) random (1) longitudinal. Modified from character 52 of Martinez et al. 2012. *Cycas*, *Stangeria*, and *Dioonopsis* all share stomata with random orientation of the guard cells.

Character 10: Thin-walled pavement cells (0) absent (1) present. Modified from character 46 of Martinez et al. 2012. Indicates the presence of thin-walled pavement cells which present a thickened cuticle.

Character 11: Pavement cell elongation (0) isodiametric (1) elongated. New character to distinguish the elongated pavement cells of most Zamiaceae from the isodiametric cells of *Cycas*, *Stangeria*, and *Dioonopsis*.

Character 12: number of accessory cell layers (0) zero (1) one/two (2) three. Modified from character 53 of Martinez et al. 2012, to acknowledge the fact that most *Macrozamia* and *Encephalartos* species present a variable number of layers (between one and two).

Character 13: Trichome bases (0) angular (1) circular. New character to distinguish the angular trichome bases found in Cycas and Dioonopsis from the circular trichome bases of the Zamiaceae.

Character 14: Eophyll (0) undifferentiated (1) differentiated. New character that includes the variation in eophyll differentiation observed in this manuscript.

Character 15: Guard cells (0) sunken (1) flush. Character 50 of Martinez et al. 2012.

References

Calonje M, Meerow AW, Griffith MP, Salas-Leiva D, Vovides AP, Coiro M, Francisco-Ortega J. 2019 A Time-Calibrated Species Tree Phylogeny of the New World Cycad Genus *Zamia* L.(Zamiaceae, Cycadales). *International Journal of Plant Sciences* **180**, 286–314.

Martinez Martínez LCA, Artabe AEE, Bodnar J. 2012 A new cycad stem from the Cretaceous in Argentina and its phylogenetic relationships with other Cycadales. *Botanical Journal of the Linnean Society* **170**, 436–458.

Salas-Leiva DE, Meerow AW, Calonje M, Griffith MP, Francisco-Ortega J, Nakamura K, Stevenson DW, Lewis CE, Namoff S. 2013 Phylogeny of the cycads based on multiple single-copy nuclear genes: congruence of concatenated parsimony, likelihood and species tree inference methods. *Annals of botany* **112**, 1263–1278.
